# Selective Autophagy in Hyperglycemia-Induced Microvascular and Macrovascular Diseases

**DOI:** 10.3390/cells10082114

**Published:** 2021-08-17

**Authors:** Leena P. Bharath, Jack Donato Rockhold, Rachel Conway

**Affiliations:** Department of Nutrition and Public Health, Merrimack College, North Andover, MA 01845, USA; rockholdj@merrimack.edu (J.D.R.); conwayre@merrimack.edu (R.C.)

**Keywords:** autophagy, cardiovascular, diabetes, endoplasmic reticulum, ER-phagy, lysosome, mitophagy, mitochondria, pexophagy, pexoxisome, reactive oxygen species (ROS)

## Abstract

Dysregulation of autophagy is an important underlying cause in the onset and progression of many metabolic diseases, including diabetes. Studies in animal models and humans show that impairment in the removal and the recycling of organelles, in particular, contributes to cellular damage, functional failure, and the onset of metabolic diseases. Interestingly, in certain contexts, inhibition of autophagy can be protective. While the inability to upregulate autophagy can play a critical role in the development of diseases, excessive autophagy can also be detrimental, making autophagy an intricately regulated process, the altering of which can adversely affect organismal health. Autophagy is indispensable for maintaining normal cardiac and vascular structure and function. Patients with diabetes are at a higher risk of developing and dying from vascular complications. Autophagy dysregulation is associated with the development of heart failure, many forms of cardiomyopathy, atherosclerosis, myocardial infarction, and microvascular complications in diabetic patients. Here, we review the recent findings on selective autophagy in hyperglycemia and diabetes-associated microvascular and macrovascular complications.

## 1. Introduction

Cardiovascular disease (CVD) affects individuals with type 2 diabetes (T2D) globally. With coronary artery disease (CAD) and stroke as major contributors to mortality, CVD is the cause of death in approximately half of the people with T2D [1]. The diseases of the coronary arteries, peripheral arteries, and cerebrovasculature are macrovascular complications promoted by diabetes. Apart from the macrovascular complications, long-term changes occur within the smaller blood vessels which promote microvascular complications such as nephropathy, retinopathy, and neuropathy. Microvascular diseases are significantly associated with a higher risk of peripheral artery disease (PAD) incidences in patients with T2D after adjusting for several established risk factors [2]. 

Autophagy is a conserved cellular homeostasis and defense mechanism that plays a pivotal role in recycling cellular materials, promoting energy, and clearing pathogens. The three known forms of autophagy are macroautophagy, microautophagy, and chaperon-mediated autophagy. While chaperon-mediated autophagy is selective and only shown to occur in mammalian systems, microautophagy and macroautophagy can be selective or non-selective, and are described in yeast and higher organisms. Macroautophagy has been extensively studied over the past decade. During macroautophagy, a double membraned autophagosome is generated which engulfs the cytosolic components. The outer membrane of the autophagosome fuses with the membrane of the lysosome or the endosome, and the contents, along with the inner membrane, are exposed to the contents of the lysosome for degradation and recycling [3]. Macroautophagy, along with chaperon-mediated autophagy and the proteosome, recycles cellular proteins. Microautophagy is classified into three types depending on the architecture of the membranes and the organelles involved in the process. Type 1 microautophagy involves the protrusion of the lysosomal membrane to capture the cytoplasmic contents, the invagination of the lysosomal membrane is type 2 microautophagy, and type 3 is the invagination of the endosomal membrane [3]. Chaperon-mediated autophagy (CMA) does not involve the formation of autophagosome or autolysosome. Proteins are degraded, but organelles are not. The protein cargo is delivered directly into the lumen of the lysosome with the aid of proteins HSC70 and LAMP-2A. The proteins that are to be degraded by CMA have at least one amino acid motif that is the penta peptide motif KFERQ. The HSC70 recognizes and forms a complex with proteins that have the pentapeptide motif. The complex interacts with the cytoplasmic tail of lysosomal LAMP2A, which promotes the delivery of the target proteins into the lumen of the lysosome. 

Autophagy is also classified into two types based on nutritional status. Non-selective autophagy occurs during nutrient starvation, and selective autophagy of organelles such as mitochondria, peroxisomes, and endoplasmic reticulum (ER) can occur in nutrient-rich conditions. A brief description of some of the key events and players in macroautophagy is discussed in the next section. The process of autophagy is reviewed in detail [4].

### 1.1. Initiation of Autophagy

Factors such as amino acid starvation [5], alterations in lipid metabolism [6], calorie restriction [7], exercise [8], impaired intracellular cholesterol transport and the presence of protein products, damaged organelles, and infectious pathogens influence autophagy [9,10,11]. Two major complexes are involved in the initiation of autophagy. The ULK1 complex and the class III PI3 Kinase complex 1. ULK1 is a complex of FIP200, ATG13, and ATG101. The post-translational phosphorylation and ubiquitination of ULK1 by ULK1 kinase is necessary for its activation. ULK1 kinase is regulated by mTORC1 and AMPK. The ULK1-ATG13-FIP200-ATG101 complex senses the nutritional status of the cells, which results in the induction of autophagy. During nutrient-rich conditions, mTORC1 associates with the ULK1 complex and phosphorylates ULK1 and ATG13. Under starvation conditions, mTORC1 disassociates, resulting in the dephosphorylation and induction of autophagy. ULK1 also phosphorylates and activates other autophagy proteins, such as Beclin1 (BCN1) and vacuolar sorting 34 protein (VPS34).

### 1.2. Nucleation 

The next complex essential for autophagy progression is the ATG14- class III phosphatidylinositol 3-kinase complex (PtdIns3K), which is involved in the nucleation of the phagophore [12]. The PtdIns3K generates PtdIns3P, which is critical for the nucleation of phagophore consisting of PIK3C3/Vps34, PIK3R4/p150, and BCN1. The complex could initiate classical macroautophagy by association with ATG14 or activate the endocytic pathway by associating with UVRAG. Further studies are required to characterize the precise role of UVRAG in autophagy. The PtdIns3K complex is regulated by proteins that interact with BCN1. The antiapoptotic protein BCL2 interacts with BCN1 and prevents its interaction with the PIK3C3, thus inhibiting autophagy. There are many positive and negative regulators of PtdIns3K complex. The positive regulators are the AMBRA1 which directly binds with BCN1, and the SH3GLB1, which can interact with BCN1 through UVRAG. These signaling events are complex and multifaceted, and further research is required to map out the upstream signaling that regulates the PtdIns3k (reviewed in [13]). 

### 1.3. Elongation

The expansion/elongation of the phagophore involves two conjugation systems with ubiquitin-like proteins. The ATG12-ATG5-ATG16L1 complex associates with the phagophore membrane but disassociates following autophagosome completion. The second system is the ATG8/LC3 system [14]. The ATG4 is a protease and is an essential component of the ATG8/LC3 system. ATG4 cleaves pro-LC3 to form LC3-I, which is lipidated to form LC3-II, thus forming the elongating phagophore membrane. Lipidation of LC3-I occurs during nutrient starvation and cellular stress. Another protein that is hypothesized to be involved in the elongation of the phagophore membrane is the ATG9. ATG9 is known to localize to the trans-golgi network and the late endosome during nutrient-rich conditions. However, under starvation conditions, the protein localizes with the autophagosome [15].

### 1.4. Lysosomal Fusion

The completion of the autophagosome and the fusion with the lysosome is the least understood process. The process is known to depend on the cytoplasmic microtubular network. The involvement of the SNARE machinery with VAM7 and VAM9 having a role in the fusion of phagosome with the lysosome is reported [16]. The localization of syntaxin 17 to the phagosome via interaction with SNAP29 and lysosomal SNARE VAMP8 is also shown to be required for the fusion [17].

### 1.5. Signaling in Macroautophagy

Many cellular events are required and play a vital role in the autophagy process. Cellular nutrient sensor and regulator mTOR is known to orchestrate major signaling events in autophagy and is thus known as the master regulator of autophagy. mTOR is also known to play pleiotropic roles, and mTOR independent autophagy is also reported [18,19]. Rapamycin and rapalogs bind to cytosolic protein FKBP12 and target a specific domain of mTOR when it is part of mTORC1 to inhibit mTORC1, which is shown to activate autophagy. mTORC2 is not sensitive to rapamycin, but prolonged treatment could cause mTORC2 inhibition [20]. Other essential regulators of autophagy include cAMP-dependent protein kinase A, cytoplasmic calcium and calmodulin-dependent kinase kinase β, AMPK, and insulin/insulin receptor signaling. It is generally hypothesized that mTOR senses changes in the nutritional status and is directly phosphorylated in response to nutrient signals. Studies also show the involvement of Ras-related small GTPases in modulating mTOR in response to amino acids [21]. The role of AMPK in autophagy initiation is also well known. Under glucose starvation, AMPK associates with and initiates ULK1. Additionally, AMPKα1 is required for autophagosome maturation and lysosomal fusion [22]. Insulin and insulin-like growth factors can also regulate mTOR through PtdIns3K. Autophosphorylation events following insulin binding promote the generation of phosphatidylinositol (3,4,5)-trisphosphate, which causes the membrane recruitment of Akt and its activator kinase PDK1. Activated Akt phosphorylates tumor suppressor gene TSC2, which prevents the formation of TSC1/2 complex. This, in turn, keeps Rheb in its active GTP bound form to phosphorylate and activate mTOR, which is inhibitory to autophagy. Ras-MEK1/2 and ERK1/2 are also known to regulate autophagy in various contexts [23]. Store-operated calcium entry is known to activate autophagy through the CAMKK2 signaling [24]. 

Apart from nutrient sensing, organelle-specific stress responses are also known to activate autophagy. Endoplasmic reticulum (ER) stress resulting from protein aggregates, hypoxia, oxidative stress, glucose deprivation, decreased energy for chaperon activity, and calcium efflux from the ER modulate autophagy. Mild or moderate stress can be an adaptive mechanism and can be beneficial. In this case, basal autophagy activation can help in the restoration of cellular homeostasis. However, prolonged or excessive stress can be detrimental. A common consequence of ER stress response is the downregulation of AKT, which in turn can upregulate autophagy by decreasing mTOR activity. The transcriptional activity of ATF6 is well established to upregulate chaperon HSPA5, which triggers autophagy by inhibiting AKT. In response to ER stress, proteins ATF4 and CEBPB can repress mTORC1 by REDD1 mediated dephosphorylation of Akt. Additionally, perturbations in calcium within the ER can activate calcium-regulated autophagy (reviewed in [25]).

**Figure 1:**
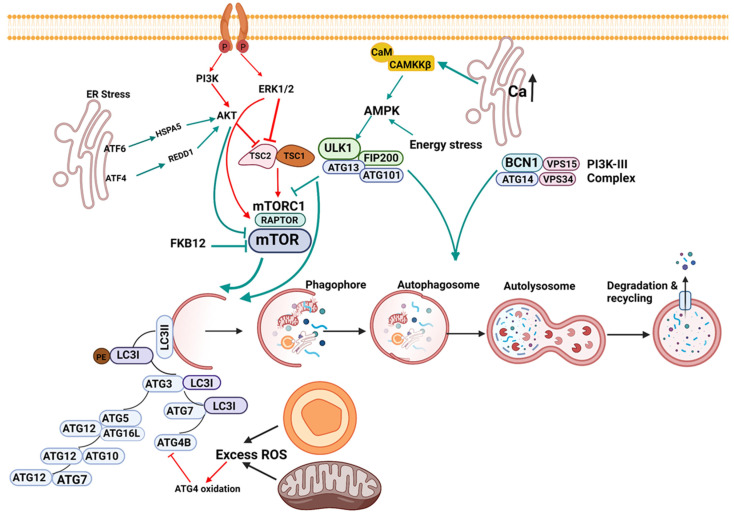
Signaling in macroautophagy. The figure depicts some of the prominent signaling pathways of autophagy. The activation of the PI3K/AKT/ERK1/2 kinases promotes the phosphorylation and activation of the mTOR pathway, thereby inhibiting autophagy. AKT activates mTOR by directly phosphorylating and inhibiting TSC2. ERK phosphorylates and functionally inactivates TSC2. ERK can also phosphorylate raptor of the mTORC1 complex and promote the activation of mTOR (red arrow). An increase in calcium within the ER, calmodulin-binding, and stress conditions such as amino acid starvation activates CAMKKβ, which activates AMPK. Energy stress is well known to activate AMPK. AMPK activates ULK1, a mammalian autophagy initiating kinase, which leads to the inhibition of mTORC1, leading to the activation of autophagy (green arrows). In addition, ULK1 can phosphorylate Beclin-1 in association with ATG14 to promote autophagy.

Similarly, stress response originating from the mitochondria and peroxisomes can also play a role in autophagy. Reactive oxygen species (ROS) regulate autophagy via multiple signaling mechanisms. Mitochondria are potent producers of cellular ROS and thus play a vital role in autophagy induction. The protease ATG4 plays an essential role in the elongation and completion of the autophagosome. The redox status of ATG4 is critical in modulating this process. While transient oxidation of ATG4 is necessary for autophagosome generation, excessive oxidation of ATG4 due to mitochondrial and peroxisomal oxidative stress can prevent the maturation of LC3 and inhibit autophagy. Additionally, thiol redox homeostasis within mitochondria, mitochondrial dynamics, and mtDNA integrity are also crucial in regulating autophagy (reviewed in [26]). Some of the prominent signaling pathways of autophagy are shown in Figure 1.

The ER domain of ATF6 interacts stably with HSPA5 in cells that are not under stress. A stress response results in the dissociation of HSPA5 from ATF6, which downregulates AKT, leading to inhibition of mTOR and the induction of autophagy. ATF4 represses mTOR by REDD1 mediated dephosphorylation of AKT. Mitochondrial and cellular ROS can activate autophagy at basal conditions. However, prolonged or exaggerated ROS can inhibit autophagy by oxidation of ATG4, a critical protein for the generation of LC3I. ATG protein network is essential for forming LC3II, which incorporates into and forms the growing phagophore. The autophagosome encloses the cytosolic components that must be degraded and fuse with lysosome, resulting in the degradation and recycling of the contents. Autophagy activation signaling is shown as green arrows, and inhibitory signaling is shown as red arrows.

Autophagy is also known to be regulated at the transcriptional level; however, transcriptional regulation of autophagy is not well understood. Transcriptional factor p53 is known to have either pro or anti autophagy effects, based on where it is localized with the cell, and thus can be a repressor or activator of autophagy [27]. Zinc Finger with KRAB and SCAN Domains 3 (ZKSCAN3) represses many lysosomal and autophagy genes in nutrient-rich conditions [28]. Akt phosphorylation induced by the insulin and growth factors regulates the transcriptional factor FOXO. FOXO3 is one of the first members of the FOXO family to be identified as a transcriptional regulator of autophagy. Transcriptional regulation of autophagy was recently reviewed in [29].

### 1.6. Selective Autophagy of Organelles

Removal of specific organelles that are either dysfunctional, damaged, or in excess occurs in the cells of organisms from yeast to mammals. The process is highly regulated and is critical for cellular homeostasis. Organelle-specific autophagy can clear mitochondria, endoplasmic reticulum, peroxisomes, lysosomes, ribosomes, and the nucleus. An initiation event, followed by highly coordinated signal transduction, is involved in organelle autophagy. Proteasome-mediated degradation and autophagy-lysosome pathways are the two major pathways by which selective organelle degradation and recycling occur. The proteasome pathway uses the 26S proteasome to degrade its substrates and removes misfolded and large aggregated proteins [30]. 

**Figure 2:**
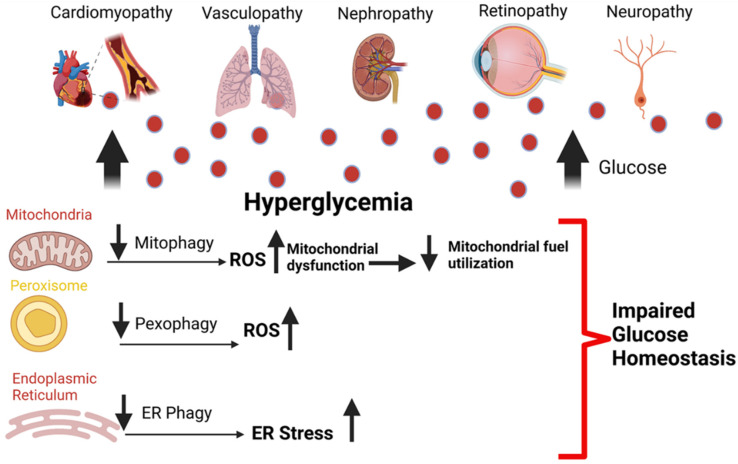
Hyperglycemia dysregulates selective autophagy and promotes diabetic complications. Hyperglycemia induced organelle dysfunction promotes cellular dysfunction. Impaired mitophagy promotes excessive mitochondrial ROS production, impaired mitochondrial energy metabolism, and fuel utilization. Peroxisomal damage results in failure of the redox system within the organelle, dysregulation of fatty acid oxidation, and impaired import of proteins. Protein misfolding within the endoplasmic reticulum (ER) causes the unfolded protein response, which induces ER stress. Hyperglycemia stalls the clearance of the damaged and dysfunctional organelles due to inefficient autophagy. Impaired fusion of the autophagosome with the lysosome, decreased lysosomal acidification, and efficiency leads to the accumulation of dysfunctional organelles, resulting in an unhealthy cellular milieu, promoting diabetic macrovascular and microvascular complications.

The initiating event for selective autophagy generally begins with the tagging of the organelle that is marked for degradation. Ubiquitination of the target organelle tags it for selective autophagy. However, ubiquitination independent signals can also initiate selective autophagy [31]. Three essential criteria must be met for selective autophagy. First, the cargo that needs to be recycled must be recognized. Second, the cargo must be pulled into the autophagosome. Third, the cellular components which are not marked for degradation must be excluded from the autophagosome. Once target organelles are tagged, adaptor proteins or cargo recognition proteins bring the tagged organelle into the phagophore. The cargo recognition proteins and degradation signals are unique for each organelle [32]. Many studies report hyperglycemia induces dysregulation of selective autophagy and promotes microvascular and macrovascular diseases (Figure 2).

## 2. Mitophagy

Mitochondria are essential for regulating cellular energy generation, homeostasis, redox signaling, programmed cell death, and immunity. Dysregulation of mitochondrial function, damage to the mitochondrial structure, or adverse changes in mitochondrial dynamics can cause diabetic complications. Mitophagy is the selective removal of mitochondria by the autophagic machinery. When mitochondria are damaged or dysfunctional, mitophagy plays a crucial role in clearing the mitochondria and restoring cellular homeostasis. There are three types of mitophagy. In type 1 mitophagy, mitochondrial fission occurs, followed by the sequestration of the mitochondria into mitophagosomes. Mitochondrial membrane depolarization does not occur until after the mitochondria are sequestered into the mitophagosome. Hence, PINK1/Parkin signaling may or may not be involved in type 1 mitophagy. PTEN-Induced Kinase 1(PINK1), is a mitochondrial serine-threonine protein kinase. PINK1 activity causes the E3 ubiquitin ligase; Parkin to localize on the depolarized mitochondrial membrane to induce mitophagy. In type 2 mitophagy, mitochondrial fission does not appear to be crucial. However, a continuous fusion of mitophagosomal membrane occurs with LC3, located on the depolarized mitochondria, consistent with the fusion of the mitochondrial membrane with the autophagosome membrane. In type 2 mitophagy, mitochondrial membrane depolarization precedes sequestration, and PINK1/parkin is involved. Type 3 mitophagy involves the formation of mitochondria-derived vesicles (MDV). The MDV undergoes fission and bud off from the mitochondrial surface. However, the budding off of the MDV does not require the participation of dynamin-related protein 1(Drp1), which is necessary for the binary fission of the mitochondria. PINK1/parkin is involved in type3 mitophagy, as the MDV is depolarized before undergoing mitophagy. The MDV contains the oxidized mitochondrial proteins, which bud off and are sequestered into vesicular bodies, which fuse with the lysosome to degrade mitochondrial contents [33].

## 3. ER-Phagy

Autophagy is also required for the elimination of the ER with unfolded and misfolded proteins. ER-phagy (reticulophagy) can be induced by ER stress. ER-phagy is necessary to prevent ER expansion-induced ER stress, and serves as a means by which misfolded proteins can be degraded [34]. ER-phagy can occur by microautophagy, where the organelle is engulfed into endosome and lysosomes for degradation. ER stress perse can also activate macroautophagy, where ER and other organelles can be sequestered into the autophagosome and delivered to the lysosome for degradation. Growing evidence suggests that the ER supplies the autophagosome membrane, and ER and autophagy are intricately connected [35]. 

## 4. Pexophagy

Although not as well characterized as mitophagy in the context of hyperglycemia, pexophagy, the selective removal of peroxisomes by autophagy machinery, is essential for overall cellular health [36]. Once considered fossil organelles, peroxisomes are now recognized to play an indispensable role in physiological and pathological conditions. Peroxisomes are found abundantly in the liver and the kidneys, and are believed to develop from endomembranes such as the endoplasmic reticulum. The peroxisome is involved in two very important tasks, fatty acid oxidation and hydrogen peroxide metabolism [37]. The three independent mechanisms that clear unwanted peroxisomes include Lon protease, 15-lipoxygenase mediated lysis, and autophagy [38].

## 5. An Overview of the Role of Autophagy in Diabetes

Type-2 Diabetes (T2D) is a metabolic disease characterized by hyperglycemia resulting from defects in insulin secretion, insulin action, or both [39]. Autophagy might be playing a pleiotropic role in T2D, and may have a context-specific role. Recent studies highlight the protective role of autophagy against hyperglycemia-induced cellular stress in pancreatic beta cells. T2D is characterized by the accumulation of islet amyloid polypeptide (IAPP). Inhibition of autophagy in cell culture aggravated the detrimental effects of IAPP. Human IAPP knockin mouse with β cell-specific autophagy defect had substantial deterioration of glucose tolerance and dispersed cytoplasmic expression of p62 associated toxic oligomers, which would otherwise be sequestered in p62-positive inclusions [40]. At the onset of diabetes, p53 accumulates in the cytosol of mouse β cells and inhibits mitophagy. Mice deficient in p53 have restored mitophagy, and are resistant to β cell reduction by streptozotocin [41]. The association between mTOR activity and autophagy and its mechanistic link to T2D induced cardiomyopathy is reported [42]. PRAS40 mediated mTORC1 inhibition prevents the development of cardiomyopathy, along with improved metabolic function, insulin sensitivity, and reduced systemic hyperglycemia in a diabetic mouse model [42]. Rapamycin, an mTOR inhibitor and inducer of autophagy, reduced body weight, heart weight, plasma glucose, triglyceride, and insulin levels in diabetic mice. Reduced oxidative stress, improved redox balance, and improvements in fractional shortening were also observed [43].

Similarly, resveratrol-induced enhancement in autophagic flux resulted in the amelioration of diabetic cardiomyopathy [44]. Breeding of β-cell-specific autophagy-deficient mice with ob/ob mice resulted in the mice developing severe diabetes, β-cell death, and oxidative stress. The inability of autophagy-deficient β-cells to manage the unfolded protein response and ER stress resulted in adverse outcomes [45]. Many animal models show that inhibition of autophagy exacerbates metabolic defects caused by hyperglycemia, obesity, and diabetes [46].

Kosacka et al., 2015, observed that human adipose tissue autophagy is upregulated in obesity and T2D, and increased autophagy occurred together with AT inflammation [47]. Higher expression of lipid droplet (LD) associated protein (PLIN2), and significant changes in lipid metabolism, apoptosis, and oxidative stress genes were observed in β cells of T2D patients. The increased LD buildup was accompanied by downregulation TFEB, a master regulator of autophagy, and the downregulation of lysosomal marker LAMP1. The findings were confirmed in the rat INS-1 cell culture model. The researchers conclude that autophagy-mediated lipid clearance and cellular homeostasis are markedly dysregulated in hyperglycemic conditions [48]. Quian et al., 2018, established the regulatory link among S-nitroglutathione reductase (GSNOR), inflammation, and autophagy in diabetes and obesity. In both mice and humans, diabetes and obesity result in nitrosative stress due to decreases in GSNOR activity. Experimental deletion of GSNOR in mice resulted in nitrosative stress, impaired hepatic autophagy, and insulin resistance. Liver-specific overexpression of GSNOR enhanced lysosomal function and autophagy, and remarkably improved insulin action and glucose homeostasis [49].

Most importantly, it is well known that highly effective anti-diabetic drugs such as metformin, rosiglitazone, and GLP-1 mimetics are also autophagy enhancers. Metformin stimulates autophagy and prevents β-cell apoptosis in in vitro lipotoxic conditions [50]. Collectively, the data show that diabetes and hyperglycemia-induced changes in autophagy, either upregulation or downregulation, can have a profound effect on cellular and organismal health. The role of β-cell autophagy in diabetes pathogenesis is reviewed in [51].

## 6. Autophagy and the Cardiovascular System

Autophagy is characterized extensively in endothelial and smooth muscle cells of the vasculature, in cardiomyocytes, and in the immune cells that infiltrate cardiovascular tissues such as the macrophages [52,53,54,55,56,57]. In recent years, an increased interest in autophagy and its role in lipid homeostasis and the cardiometabolic syndrome has led to the identification of new players, pathways, and therapeutic targets [49,58,59]. While many studies have shown the correlative and causative relationship among diabetes, CVD, and autophagy, the mechanistic links between autophagy and the many pathologies remain elusive. This review summarizes the recent findings on selective autophagy of organelles and their role in developing microvascular and macrovascular complications associated with hyperglycemia and diabetes. Particular emphasis is on autophagic clearance of mitochondria, as mitochondria are extensively investigated in the context of cardiovascular diseases and diabetes.

Baseline cardiovascular autophagy is a homeostatic mechanism that is elemental for maintaining cardiovascular physiology and function. Autophagy is induced and increases above the basal levels by several forms of stress such as energy deprivation, hypoxia, redox imbalance, hormones, organelle induced stress such as endoplasmic-reticulum and mitochondrial stress, oxidized lipids, danger-associated molecular patterns, and microbial stress caused by pathogen-associated molecular patterns. Autophagy is also essential for survival under nutrient deprivation conditions immediately post-birth. This was demonstrated in neonatal mouse models, which showed immediate upregulation of autophagy upon birth. Autophagy was maintained at high levels for 3–12 h post-birth and returned to basal levels within 1–2 days. Conversely, mice deficient in Atg5, a protein critical for autophagosome formation, died within a day of birth. Atg5 deficient mice were energy depleted and had a low concentration of amino acids in plasma and tissues [60]. 

Autophagy intersects the nutrient sensing and energy regulatory pathways, and aging, or disease-associated dysregulation of autophagy, has profound detrimental effects on cellular health. Evidence also points out that the decline in autophagy with age promotes CVD. One primary mechanism proposed for CVD development with the decrease in autophagy is that aged cardiomyocytes have dysfunctional mitochondria, producing less ATP, but exaggerating reactive oxygen species (ROS) [61]. Due to their postmitotic state, these cardiomyocytes do not proliferate. Therefore, they can neither dilute the dysfunctional mitochondria nor clear the mitochondria by autophagy, resulting in functional failure of cardiomyocytes leading to CVD [62,63]. Autophagy decline is observed in hypertrophied myocardium, and autophagy enhancer rapamycin prevents cardiac hypertrophy by inducing autophagy-regulating proteins Noxa and Beclin1 [64]. Accumulation of proteins due to decline autophagy is associated with cardiac remodeling, contractile dysfunction, and hypertrophy, leading to heart failure. Rapamycin treatment of mice receiving transverse aortic constriction (TAC), showed that TAC induced accumulation of modified proteins and rapamycin prevented heart failure [65]. 

Kanamori et al., 2011, assessed the effects of autophagy manipulation on acute myocardial infarction in vivo. Autophagy was activated within 30 min of coronary ligation, and pharmacological inhibition of autophagy resulted in a significant increase in infarct size. Interestingly, attenuation of infarct size was observed in mice with upregulation of starvation-induced autophagy before the coronary ligation. While inhibition of autophagy resulted in reduced postinfarction ATP levels, starvation increased myocardial amino acids and ATP levels [66]. The effects of autophagy enhancer trehalose was recently evaluated in a mouse model with left anterior descending artery ligation (LAD). Trehalose administration enhanced autophagy, improved left ventricle function, lung congestion, cardiac remodeling, apoptosis, and fibrosis in the wild-type mouse. All of the trehalose-induced beneficial effects were blunted in Beclin heterozygous mice, indicating that the beneficial effects were secondary to improvements in autophagy. While Beclin activation is associated with autophagy enhancement, ROS-mediated Beclin activation was shown to inhibit lysosomal protein LAMP2. This could probably be due to hyperactivation of Beclin1. Hypoxia-reoxygenation injury is accompanied by ROS-induced Beclin 1 upregulation but downregulation of lysosome-associated membrane protein 2 (LAMP2). Cell death induced by hypoxia-reoxygenation was attenuated by the restoration of LAMP2 via partial Beclin-1 knockdown to promote autophagosome processing [67]. The role of autophagy in cardiovascular disease models is reviewed in [68].

Similar to cardiac autophagy, vascular autophagy also dwindles with age in both mouse models and humans [54,69]. Experimental disruption of autophagy in the vascular smooth muscles of adult mice resulted in defects in calcium homeostasis and impairments in contractility and vascular reactivity [70]. Mice with experimental endothelial cell-specific disruption of autophagy had normal vascular architecture and capillary density, but displayed abnormalities in the synthesis and release of von Willebrand factor (vWF) and had prolonged bleeding times [71].

While autophagy is a beneficial response, excessive autophagy can undoubtedly be detrimental. One such mechanism is autophagy-dependent cell death, known as autosis. Autosis is characterized by numerous autophagosomes and autolysosomes, along with distinctive features such as nuclear membrane convolution and shrinkage, causing the formation of perinuclear space and endoplasmic reticulum dysmorphia. Autosis is known to promote myocardial injury [72]. Autosis is induced by autophagy-inducing peptides, starvation, and neonatal cerebral ischemia [73]. Autosis can be rescued by autophagy inhibitors, but not by inhibitors of apoptosis or necrosis, thus showing that autosis is not independent of autophagy. Experimental disruption of Beclin1 in cardiomyocytes, reduced ischaemia/reperfusion (I/R)-induced autophagy and prevented cell death. Autophagy was also inhibited by endogenous cardiac peptide; urocortin. Urocortin is known to inhibit Beclin 1 and prevent cardiomyocyte cell death [74]. These results show that Beclin 1 might play a context-specific role, and activation of the protein might be detrimental or beneficial, depending on the context and the tissue or cell type. Thus, autophagy lingers in the Goldilocks zone, where too little or too much can cause detrimental effects. 

## 7. Mitophagy and Microvascular Diseases

### 7.1. Diabetic Nephropathy

**Figure 3:**
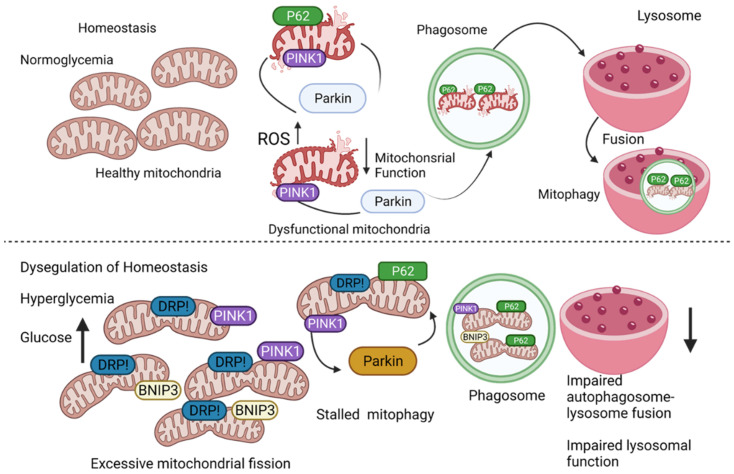
Mitochondrial homeostasis is altered by hyperglycemia.

Diabetic nephropathy (DN) is a severe outcome of the disease and the single leading cause of end-stage kidney disease. The inability to remove and recycle damaged and dysfunctional mitochondria is well established as the cause that promotes the pathogenesis of acute and chronic diabetes-induced kidney diseases such as DN. Mitochondria are highly dynamic organelles, and there exists an intricate link among mitochondrial dynamics, mitochondrial function, and mitophagy. Studies in animal models show that alterations in mitochondrial biology leading to dysfunction, play a significant role in the development of DN. Proximal tubal epithelial cells of the renal cortex are important for the reabsorption of fluids, glucose, and amino acids. As these cells have abundant mitochondria and rely on oxidative metabolism for energy generation, they are often susceptible to the functional consequences of mitochondrial dysfunction. A defect in the electron transport chain and the resulting reactive oxygen species (ROS) generation promotes microinflammation and propagates the advancement of DN [75]. Likewise, loss of mitochondrial quality control directly impacts microvascular function in the kidneys [76]. Fragmented mitochondria accumulate in both animal models and humans with DN (Figure 3). 

In normoglycemic conditions, basal mitophagy clears the mitochondria that are dysfunctional and restores homeostasis. In hyperglycemic conditions, mitochondria (i) undergo excessive fission, and (ii) are engulfed into autophagosome, but the autophagosome fail to fuse with the lysosome to complete the process; autophagosomes within the lysosomes are not degraded due to dysfunctional lysosomes. Hyperglycemia-induced mitophagy disruption promotes redox imbalance, mitochondrial dysfunction, impaired cellular metabolism, and inflammation, which can collectively promote cardiovascular diseases.

Renal ischemia-reperfusion (IR) is an established cause of acute kidney injury (AKI). Impairment in mitophagy is implicated in the onset of AKI. Tang et al. 2016, showed that mitophagy is induced in renal proximal tubular cells in both in vitro and in vivo models of ischemic AKI. Abrogation of PINK1 and Parkin 2, proteins involved in mitophagy-promoted mitochondrial damage, ROS production, and inflammatory responses, resulting in AKI [77]. Similarly, knockdown of proteins Atg5 and Atg7, involved in the elongation and expansion of the isolation membrane of the autophagosome, sensitizes kidneys to ischemia-reperfusion injury [78]. 

The occurrence of defective mitophagy, exaggerated mitochondrial ROS (mtROS) generation, and reduced mitochondrial potential was confirmed in the glomerulus of diabetic db/db mice. Dysregulated glucose homeostasis-induced effects were partially reversed by oral administration of CoQ10, which upregulated mitophagy [79]. The renoprotective effects of CoQ10 were prevented by mitophagy inhibitor Mdivi-1 or genetic inhibition of mitophagy by PINK1 knockdown. Morphological changes, such as tubular epithelial disruption, glomerular hypertrophy, and increased mesangial matrix, were not alleviated by CoQ10 in the absence of mitophagy. The study showed that the Nrf/ARE pathway was essential for CoQ10 induced renal mitophagy. To evaluate if mitophagy activation, independent of CoQ10, is renoprotective, mitophagy activator Torin was utilized. Treatment with Torin, activated mitophagy, promoted positive morphological changes in the glomeruli and reduced apoptosis, evidenced by reduced levels of c-Caspase-3, Bax/Bcl2, and cytosol cytochrome C. Diabetic nephropathy was exacerbated, and Torin was not protective when PINK1/Parkin mediated mitophagy was inhibited. Collectively, these data underscore the importance of mitophagy in renal health. In another study, chronic hyperglycemia activated nuclear receptor subfamily 4, group A, member 1 (NR4A1) in patients with DN. NR4A1 activates p53, which then selectively stimulates mitochondrial fission and inhibits mitophagy. Genetic inhibition of NR4A1, prevented mitochondrial fission factor (mff) related fission, restored Parkin-mediated mitophagy, prevented hyperglycemia-induced mitochondrial damage, all resulting in improved renal function [80].

Interestingly, some studies implicate diabetes-induced excessive mitophagy in causing kidney injury and DN. Diabetic (db/db) mice showed overt renal injury; higher expression of mitochondrial fission proteins, Dynamin-related protein (Drp1), mitochondrial fission protein (Fis-1), and mitochondrial fission factor (MFF); and abnormal activation of PINK-1/Parkin mediated mitophagy. Renal injury was prevented, along with amelioration of the progression of diabetic nephropathy, when a mitochondrial quality control network was restored by treatment with Astragaloside [81].

### 7.2. Diabetic Retinopathy

Mitophagy dysregulation is also reported in diabetic retinopathy (DR). DR is the leading cause of blindness in the working-age population. Hombrebueno et al. 2019, demonstrated the mechanistic link between dysregulated mitophagy and the progression of diabetic retinopathy. Mitochondrial biogenesis and mitophagy were evaluated in the retinas of Ins2^Akita/+^ and mitophagy reporter mice of the same background. Mild neurovascular stress-induced mitophagy resulted in the accelerated clearance of the mitochondria. This event was followed by severe neurovascular stress, which impaired mitophagy, resulting in the accumulation of damaged mitochondria [82]. Diabetes accelerated mitophagy in the outer retina of the mice. Higher PINK1 mediated mitophagy, coupled with the lack of concomitating changes in mitochondrial biogenesis, was identified as the cause of lower mitochondrial content in two-month-old diabetic mice during the early stages of the disease [82]. Mitochondrial biogenesis machinery was impaired, as evidenced by substantial mtDNA damage, decreased TFAM and mtDNA copy number. Significant decrease in mitolysosome, higher PINK1 stabilization on the mitochondria, and substantial increase in Parkin suggested that PINK1 primed mitochondria are not cleared by mitophagy in later stages of diabetes. Interestingly, impairment of mitophagy in the later stages of the disease promoted senescent retinal phenotype. Similarly, biogenesis failed to compensate for hyperglycemia-induced mitophagy in cultured retinal Mueller cells [83,84]. 

The involvement of thioredoxin-interacting protein (TXNIP) in DR was reported [85]. Mueller cells maintained at HG conditions have higher expression of TXNIP, along with mitochondrial fragmentation, loss of membrane potential, and higher ROS. Compared to cells treated with low glucose, cells treated with high glucose had higher mitophagy. CRISPR/Cas9 mediated knockout of TXNIP, preventedHG-induced mitophagy. Similar to the findings in the cell culture, TXNIP expression was higher in the retinas of diabetic rats. Upregulated TXNIP, mitochondrial dysfunction, and dysfunctional mitophagy were also observed in human retinal pigment epithelial cell lines. During the early stages of diabetes, retinal neurons are damaged, resulting in Muller cell gliosis, mitochondrial stress and depolarization, and large amounts of ROS in the retina, all of which contribute significantly to the pathology of DR.

### 7.3. Diabetic Neuropathy

Diabetes is known to affect neurons and cause neuropathy. The nerve damage that occurs in individuals with diabetes is termed diabetic neuropathy. The most common type is peripheral neuropathy, which typically affects the hands and feet of one-third to one-half of individuals with diabetes. Chronic high glucose levels are associated with a progressive rise in oxidative stress levels, mitochondrial damage in the neurons, and the promotion of diabetic neuropathy. It is also known that mutations of proteins involved in mitochondrial turnover promote diabetic neurological diseases [86]. Cerebral microvascular complications can occur as the result of endothelial dysfunction. Bovine brain microvascular endothelial cells cultured in high glucose conditions had higher oxidative stress and apoptosis. Treatment of the hyperglycemic cells with brain-derived neurotrophic factor (BDNF), promoted HIF1α/BNIP3 mediated mitophagy and prevented high-glucose induced damage [87]. Although studies using animal models in understanding the regulatory role of mitophagy on the development of diabetes-induced neuropathy is not comprehensive, in vitro studies have the connection between neuropathy and mitophagy in the context of high glucose. 

High-glucose treatment of human neuroblastoma cells induced PINK1 and LC3B expression, decreased cytochrome C oxidase subunit 4 expression, and mitotracker fluorescence, indicating loss of mitochondria mass via the activation of mitophagy. When mitophagy regulator PINK1 was silenced, mtROS and cleaved caspase 3 expression were induced, along with the accumulation of membrane potential impaired mitochondria within the cells. The authors conclude that mitophagy induction is essential for preventing neuronal cell apoptosis in high glucose conditions [88]. The role of thioredoxin interacting protein (TXNIP) on PINK1/Parkin was evaluated in PC12 cells under high glucose conditions [89]. High glucose treatment-induced TXNIP expression promoted ROS and cell death. Expression of both PINK1 and Parkin was lower, along with decreased colocalization of mitochondrial protein COX IV with lysosomal protein LAMP1, all indicative of stalled mitophagy. Overexpression of TXNIP, downregulated PINK1 and Parkin, but a knockdown of TXNIP, reversed high glucose-induced ROS production and promoted COX IV colocalization with LAMP1, indicating upregulation of mitophagy. 

## 8. Mitophagy and Macrovascular Diseases

### 8.1. Diabetic Vasculopathy

A group of debilitating conditions such as sudden rupture of blood vessels, blockage of blood flow to the brain resulting in hemorrhage, and ischemia are referred to as a stroke. Diabetes is a well-established risk factor for stroke. Diabetic patients are at a higher risk for stroke, along with poorer post-stroke outcomes. It is also important to note that 65% of diabetic patients will die of thrombotic events, including heart attack and stroke [90]. Mitochondrial dysfunction and exaggerated ROS are implicated in the development of endothelial cell dysfunction during diabetes. Endothelial cells are the single layer of cells that line the blood vessels. Endothelial cell dysfunction is often the underlying cause for the development of stroke. While some studies show that mitophagy induction in endothelial cells is beneficial in preventing the harmful effects of hyperglycemia, unchecked mitophagy can be detrimental. Hypermitophagy can promote endothelial cell damage and lethal cardiomyopathy [91]. 

In vitro cell culture studies in human umbilical vein endothelial cells show that high glucose induces functional impairment of the mitochondria, along with fragmentation and excessive ROS production. High glucose insult blunts mitophagy, thereby resulting in the accumulation of damaged mitochondria, all of which can led to endothelial cell dysfunction and cardiovascular disease onset. Diabetic mice aortas had defective mitophagy, which coincided with mitochondrial dysfunction. Infusion with mesenchymal stem cells (MSC) restored PINK1/Parkin-mediated mitophagy and prevented endothelial cell apoptosis, thereby restoring endothelial cell function [92].Inducing mitophagy in diabetic platelets is a potential treatment for alleviating excessive oxidative stress associated with hyperglycemia. Chronic high blood glucose-induced severe oxidative stress causes platelet damage in diabetic patients. Ultra-structure analysis of platelets by electron microscopy showed autophagosomes and autolysosomes in diabetic platelets, which indicate an upregulation of autophagy. A low level of oxidative stress was necessary to induce mitophagy by the JNK pathway, and the platelets were protected from exaggerated ROS by the induction of mitophagy. Mitophagy induction in both murine and human platelets prevented phosphorylation and activation of p53, promoted the removal of dysfunctional mitochondria, and prevented apoptosis, thereby reducing the risk of thrombosis [93].

### 8.2. Diabetic Cardiomyopathy

Diabetic cardiomyopathy (DCM) poses a significant morbidity and mortality risk. Dysfunctional and damaged mitochondria contribute to substantial levels of ROS in diabetic myocardium. While antioxidant treatment in vitro seems promising, studies in animals and humans fail to deliver the same benefits observed in in vitro cell culture studies. The primary reason for the failure to recapitulate the in vitro results is due to the continued presence of damaged mitochondria that generate ROS. Hence, activation of mitophagy could be a valuable therapeutic intervention for diabetic cardiomyopathy. When mitophagy was promoted by Parkin overexpression, the high-glucose-induced cardiac injury was prevented, whereas knockdown had the opposite effect [94]. Rat ventricular cardiomyocytes treated with 30mM glucose had reduced the mitochondrial connectivity and increased the number of mitochondria, which together indicate mitochondrial fragmentation. When mitochondrial fragmentation protein Drp1 was knocked down, mitochondrial fragmentation was prevented, but it did not prevent high glucose toxicity. Interestingly, when Drp1 was overexpressed, high glucose induced cardiomyocyte injury was prevented. The study also showed that Parkin overexpression increased mitochondrial fission, whereas Drp1 overexpression increased mitophagic flux, suggesting that interventions promoting mitochondrial fission and mitophagy, without aberrant activation of the processes, can be therapeutic in mitigating diabetic cardiomyopathy. Drp1 regulates mitochondrial function by altering its phosphorylation status as well as modulating the Ca^2+^ release-activated calcium channel protein 1; Orai1. High-fat diet fed Zucker diabetic rats developed cardiac hypertrophy and impaired cardiac function, along with alterations in mitochondrial dynamics and calcium handling proteins [95]. Drp1 inhibitor, mitochondrial division inhibitor 1 (Mdivi-1), prevented hyperglycemia-induced cardiomyocyte hypertrophy by reducing phosphorylation of Drp1 at serine 616 (S616) and inducing phosphorylation at serine 637 (S637). Calmodulin-binding catalytic subunit A (CnA) inhibitor, and p-ERK1/2 inhibitor also improved high-glucose-induced cardiomyocyte hypertrophy by promoting S637 and inhibiting S616 Drp phosphorylation. Increased Drp1 acetylation at lysine 642 (K642) was reported in high-fat-fed mouse hearts and cardiomyocytes incubated with palmitate. The non-acetylated Drp1 mutation (K642R) prevented palmitate-induced Drp1 activation, mitochondrial fission, contractile dysfunction, and cardiomyocyte death [96].

A recent study by Tong et al., 2019, showed that mitophagy is essential for maintaining cardiac function during diabetic cardiomyopathy [97]. Fatty acids are utilized as a fuel source in diabetic hearts, which generates high ROS levels and leads to mitochondrial dysfunction. Homeostatic mechanisms of mitophagy and mitochondrial turnover are needed to preserve the function of the heart. A mouse model of high-fat diet (HFD) induced obesity was utilized to evaluate autophagy and cardiovascular function. Cardiac autophagy was activated and peaked at 6 weeks of HFD consumption. Autophagy activation could be an adaptive response to deal with lipotoxicity. Mitophagy was evaluated utilizing mice expressing cardiac tissue-specific reporter Mito-Keima. Parkin and Atg7 dependent mitophagy was activated as early as three weeks into a high-fat diet and protected the heart against cardiac hypertrophy, diastolic dysfunction, and lipid accumulation. Deletion of Atg7 impaired mitophagy, increased lipid accumulation, exacerbated diastolic dysfunction, and induced systolic dysfunction. Mitophagy activation by Tat-Beclin1 (TB1), decreased lipid accumulation and improved mitochondrial function along with improvements in cardiac diastolic function during high-fat feeding. A similar mechanistic link between mitochondrial quality control and diastolic function was observed in pre-diabetic male rats [98].

Recently, the role of Bromodomain containing protein 4 (BRD4) on diabetic cardiomyopathy was evaluated. BRD4 is a transcriptional and epigenetic regulator which is mechanistically linked to PINK1-Parkin mediated mitophagy in diabetic cardiomyopathy. Higher BRD4 and lower PINK1 protein expression was observed in a mouse model of high-fat diet-induced diabetic cardiomyopathy. Higher mitochondrial content was observed, while mitochondrial biogenesis factors PGC1α and Tfam were lower in mice on HFD. The data from this study shows that HFD induced inhibition of PINK1/Parkin mitophagy promotes mitochondrial dysfunction and accumulation of damaged mitochondria. BRD4 inhibition by JQ1 promoted mitophagy, mitochondrial function and alleviated HFD-induced diastolic and systolic dysfunction. JQ1 effects on cardiac function were evident in wild-type mice, while it failed to restore normal cardiac functions in PINK1^−/−^ mice [99]. Next, mitophagy upregulation by supplementation with glycine was beneficial in attenuating diabetic cardiomyopathy [100]. High glucose-treated H9C2 cells were more susceptible to hypoxia/regeneration-induced injury, promoted by blunted autophagy, mitochondrial damage, oxidative stress, and PKCβ2 activation. Treatment of cells with glycine; a fundamental component of cellular redox regulator glutathione, suppressed the high-glucose induced downregulation of mitochondrial proteins mitofusion 2 and LC3II, resulting in the reactivation of PINK1/Parkin dependent mitophagy. Restoration of mitochondrial membrane potential and prevention of hypoxia/regeneration induced cellular injury were also observed. These recent findings showing a strong connection between mitophagy and cardiovascular function imply that therapeutic augmentation or modulation of mitophagy can be valuable in treating or preventing diabetic cardiovascular complications.

## 9. ER-Phagy and Vascular Complications

**Figure 4:**
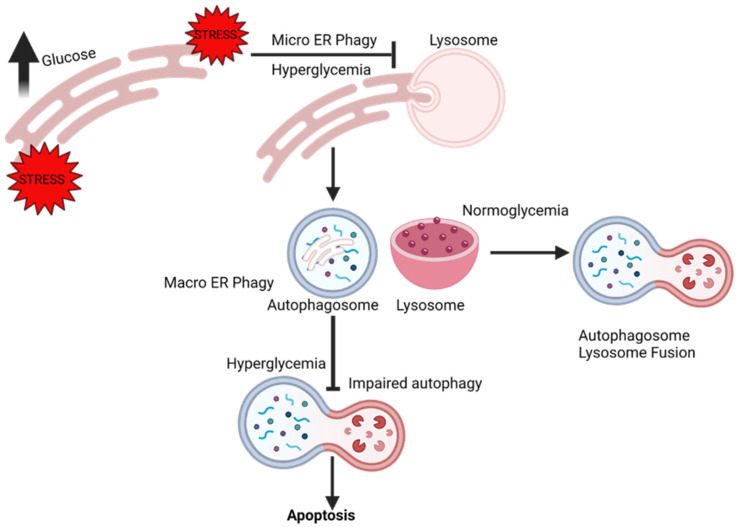
Chronic hyperglycemia disrupts endoplasmic reticulum (ER) homeostasis. When disruption of homeostasis occurs within the ER, the ER initiates the adaptive ER-stress responses such as the unfolded protein response (UPR) and autophagy. Lysosomes engulf portions of damaged ER during micro ERphagy, whereas the autophagy machinery is involved in macro ERphagy. Activation of the adaptive stress responses alleviates stress and restores homeostasis. However, unresolvable UPR activation due to chronic hyperglycemia exhausts the ER stress response, impairs autophagy, and promotes apoptosis.

It is well established that hyperglycemia and reactive oxygen species are drivers of ER stress. Therapeutic strategies to enhance ER autophagy are considered a viable option to relieve the cytotoxic stress from the ER. Fang et al., 2013, elucidated the relationship between diabetes-induced ER stress and autophagy, and the implications it has on the development of DN [101]. First, the authors observed that high glucose treatment of podocytes inhibited basal autophagy and caused podocyte injury in autophagy-dependent manner. Second, high glucose exhausted ER stress response and promoted apoptosis (Figure 4). Third, pharmacological intervention that prevented the phosphorylation of ER stress response protein eIF2α restored autophagy and induced podocin production in high glucose conditions, showing that prolonged hyperglycemia can exhaust the cytoprotective output of ER stress, which might be essential for the autophagic flux. Next, the effect of taurine-conjugated deoxycholic acid (TUDCA) was evaluated. TUDCA enhanced the cytoprotective capacity of ER and autophagy. TUDCA, along with improving autophagy and promoting the remission of ER stress, improved mesangial matrix expansion, basal membrane thickening, podocin expression, and ameliorated urine albumin levels.

While autophagy can have beneficial effects, the dual role of autophagy is reported in DR. Cultured human retinal capillary pericytes were treated with heavily oxidized glycated (HOG-LDL), or HOG-LDL was injected intravitreally to streptozotocin-induced diabetic mice. The mice were double knockouts for LDL receptor and apolipoprotein B to model hypercholesteremia. HOG-LDL elicited ER stress and autophagy and caused significant cell death. HOG-LDL induced effects were prevented, along with improvements in cell survival, when autophagy was inhibited [102]. As mentioned above, it is now known that aberrant activation of autophagy can be as detrimental as autophagy dysregulation, and it is context- and cell-type-specific.

The G-protein coupled receptor AJP and its ligand apelin are expressed in many tissues including the adipose tissue, skeletal muscles, and the central nervous system [103]. Studies in humans reveal that a high level of apelin is beneficial and decreases the risk of diabetes [104]. However, recent studies show that apelin-13 induced cardiomyocyte hypertrophy had connections to ER stress and autophagy. Invitro treatment of cardiomyocytes with apelin-13, promoted the expression of ER stress proteins Bip and CHOP, and increased the formation of autophagosomes, ER fragments, and LC3 puncta on the ER of the cells. Genetic inhibition of APJ, Bip, CHOP, and NOX4 or pharmacological inhibition of NADPH prevented apelin-13 induced overexpression of LC3 and Beclin, and suppressed cell diameter, volume, and protein content, thus preventing hypertrophy [105]. Altogether, these findings underscore the importance of ERphagy in maintaining cardiovascular health.

## 10. Pexophagy and Vascular Complications

**Figure 5:**
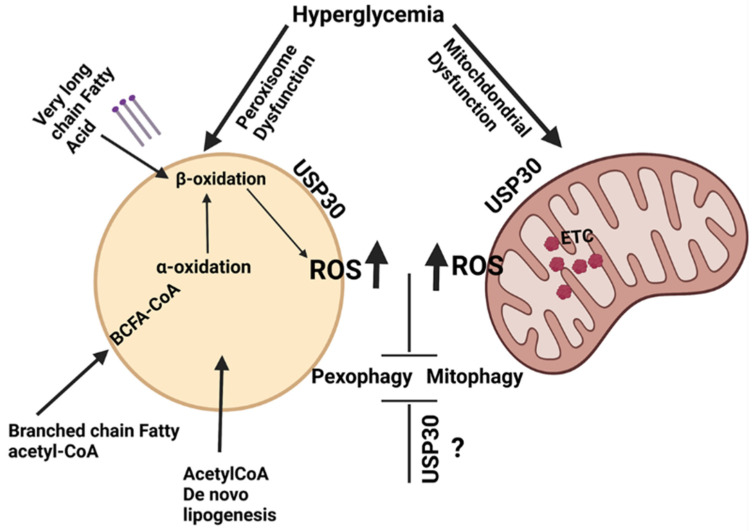
Suppression of pexophagy and mitophagy promotes exaggerated oxidative stress in hyperglycemic conditions.

Peroxisomal ROS formation is implicated in the onset of DN [106]. Oxidative damage and endothelial cell peroxisomal dysfunction occur in response to acute lipopolysaccharide-induced kidney damage [37]. Vasko et al. demonstrated that activation of pexophagy is a default response to endotoxic stress. Impaired pexophagy, accompanied by lysosomal dysfunction, often occurs in chronic diseases such as diabetes. The appropriate or hormetic concentrations of ROS promote autophagy and aid in the clearance of organelles to restore cellular health, whereas high ROS damages organelles, including the mitochondria and the peroxisome. Dysfunctional peroxisomes not recycled by autophagy can be organelles of significant cellular ROS. Higher levels of free fatty acids and lower ATP levels are observed in ischemic kidneys. Ischemia is known to impair renal fatty acid oxidation, not only in the mitochondria, but also in peroxisome. It is evident from the available literature that dysregulated ROS homeostasis due to inadequate pexophagy is instrumental in promoting renal vascular complications in diabetes. Hence, appropriately activated pexophagy is essential for kidney homeostasis [37]. Dysregulation of pexophagy can also promote complications such as diabetic retinopathy. The chronic state of hyperglycemia during the early stages of DR can cause damage to the microvasculature, and promote increased vascular permeability and occlusion [107]. As DR advances, retinal ischemia and hypoxia develop, resulting in oxidative stress, inflammation, ER stress, and accumulation of advanced glycation end products (AGEs), which can cause retinal neovascularization, which in turn can cause vitreous damage, retinal detachment, and visual disability. As peroxisomes are organelles of ROS control and fatty acid oxidation, a dysregulation of their function, along with impaired clearance of the dysfunctional peroxisome, can rapidly promote factors that promote DR. Recently, the role of USP30, an integral mitochondrial outer membrane protein, in pexophagy was reported [108]. USP30 is a deubiquitylase, which counters Parkin-dependent mitophagy by deubiquitylating the outer mitochondrial membrane proteins, especially TOMM20. USP30 regulates basal mitophagy in a PINK1 dependent (but not parkin) mitophagy. A different pool of USP30 is on peroxisomes, and inhibits basal pexophagy without the requirement of PINK1. The data point to the dual role of USP30 in tonically suppressing both pexophagy and mitophagy. It would be interesting to evaluate the if dual suppression of pexophagy and mitophagy by USP30 occurs in diabetes and its functional implications in CVD (Figure 5).

The hyperglycemia/diabetic condition is known to induce peroxisomal and mitochondrial dysfunction. In combination, the ROS generating metabolic processes occurring within the peroxisome and the mitochondria can exaggerate oxidative stress, possibly initiating a cycle of events that in turn suppresses autophagy. Activation of deubiquitylase USP30 is known to suppress both pexophagy and mitophagy. Further research is needed to evaluate the precise role of USP30 in diabetes and CVD.

## 11. Crosstalk and Organelle Autophagy

Dysfunction of multiple organelles occurs during metabolic diseases such as diabetes. Quality control of organelles is essential for maintaining the integrity of the cell. Therefore, the crosstalk among the organelles and the quality control process is necessary. In general, the autophagy machinery uses the ATG proteins across the different forms of organelle autophagy. The completion of the process requires the lysosome and associated proteins, which is also a shared process. However, specific cargos are recognized by special signals, and are pulled into the autophagosome by adaptor proteins. The adaptor proteins can be unique to the organelle, or shared among the different organelles. It is known that the ER and the mitochondria maintain close contact. ER and the mitochondria exchange messages by the physical association between the organelles through the mitochondria-associated membranes (MAMs). The MAMs are shown to be involved in the regulation of autophagy. Hamasaki et al., 2013, demonstrated that, under starvation conditions, the translocation of ATG4 to MAMs increases, and ATG5 translocate to the MAMs until the autophagosome is formed. This indicates that an intricate cell signaling and crosstalk must exist between the organelles and the autophagy machinery [109]. A direct relationship between the MAMs and FUNDC1 mediated mitophagy was also reported, showing a signal transduction pathway involving the mitochondria, the ER, and the cytosol [110]. Further research is needed to elucidate the crosstalk among the organelles and the autophagy machinery, the initiating events, and the signaling pathways that facilitate the crosstalk.

## 12. Conclusions

In recent years, evaluating the role of selective autophagy in cellular health is possible due to the advancements in molecular techniques, microscopy, and targeted probes. Mitochondria-targeted probes are utilized to visualize and monitor mitochondrial dynamics, function, and mitophagy. The field of cardiovascular biology has used these probes extensively. Electron microscopy, fluorescently tagged probes, probes that emit fluorescence at different spectra according to their cellular location, such as mitoTimer, mt-Keima, and transgenic mouse models expressing fluorescent probes such as mito-QC mouse, reveal precisely the biochemical mechanisms at cellular and organismal levels. Although much is known, especially about mitophagy, much remains to be explored regarding the precise role of mitophagy in the context of health and diseases. However, there is consensus that hyperglycemia-induced dysregulation of mitophagy can adversely affect overall health and promote CVD. In certain contexts, it is beneficial to promote mitophagy, whereas, in others, it is necessary to prevent excessive mitophagy. It is essential to develop therapeutics that would alter the process appropriately to maintain it in the “Goldilocks zone.” It is also necessary to develop therapeutics targeted to specific organelle (i.e., mitochondria or ER) to minimize off-target effects and modulate organelle-specific processes towards the goal of promoting health. Our understanding of selective autophagy of organelles such as the peroxisomes and ER in the context of diabetes and CVD is in its nascent stages. However, over the past decade, many new pathways, proteins, and mechanisms were identified, which will enable us to understand these processes in physiological and pathological contexts. These efforts will lead to new avenues of research, therapies, and lifestyle interventions to delay or prevent the onset of cardiovascular diseases.

## Abbreviation

AGEs	Advanced glycation end products
AKI	Acute kidney injury
Akt	Protein kinase B
AMBRA 1	Activating molecule in BECN1-regulated autophagy protein 1
AMPK	AMP-activated protein kinase
AMPKα1	AMP-activated protein kinase alpha-1
APJ	Apelin receptor
ATF4	Activating Transcription Factor 4
ATF6	Activating Transcription Factor 6
ATG4	Autophagy related protein 4
Atg5	Autophagy related protein 5
Atg7	Autophagy related protein 7
ATG8/LC3	Autophagy-related protein 8
ATG9	Autophagy-related protein 9
ATG13	autophagy related protein 13
ATG14	autophagy related protein 14
ATG101	autophagy related protein 101
ATP	Adenosine triphosphate
BCL2	B-cell lymphoma 2
BCN1	Beclin 1
BDNF	Brain-derived neurotrophic factor
BNIP3	BCL2 Interacting Protein 3
BRD4	Bromodomain containing protein 4
Bax	bcl-2-like protein 4
Bip	Binding immunoglobulin protein
CAMKK2	Calcium/Calmodulin Dependent Protein Kinase Kinase 2
cAMP	Cyclic adenosine monophosphate
C-caspase-3	Cleaved caspase-3
CAD	Coronary artery disease
CEBPB	CCAAT Enhancer Binding Protein Beta
CHOP	C/EBP homologous protein
CMA	Chaperon-mediated autophagy
CoQ10	coenzyme Q10
COX IV	mammalian cytochrome c oxidase complex five
CRISPR-Cas9	clustered regularly interspaced short palindromic repeats protein 9
CVD	Cardiovascular disease
DCM	Diabetic cardiomyopathy
DN	Diabetic nephropathy
DR	Diabetic retinopathy
Db	Diabetic
Drp1	Dynamin-related protein
eIF2α	Eukaryotic Initiation Factor 2
ER	Endoplasmic reticulum
ER-phagy	Endoplasmic reticulum reticulophagy
ERK1/2	Extracellular signal-regulated kinases
Fis-1	Mitochondrial fission protein
FIP200	FAK family kinase-interacting protein of 200 kDa
FKB12	FK506-binding protein 12-rapamycin-associated protein 1
FOXO	Forkhead box
FOXO3	Forkhead box O3
GLP-1	Glucagon-like peptide 1
GTP	Guanosine triphosphate
GTPases	GTP binding protein
GSNOR	S-nitrosoglutathione reductase
H9C2	Cell line
HFD	High fat diet
HG	High glucose
HIF1α	Hypoxia Inducible Factor 1α
HOG- LDL	High oxidized glycated low density lipoprotein
HSC70	Heat shock cognate 71 kDa protein
HSPA5	Heat Shock Protein Family A Member 5
IAPP	Islet amyloid polypeptide
IR	Ischemia-reperfusion
Ins2 Akita/+	mouse model
JNK	c-Jun N-terminal kinase
JQ1	Bromodomain and extra-terminal domain inhibitor
LAMP1	lysosome-associated membrane glycoprotein 1
LAMP-2A	Lysosome-associated membrane protein 2
LC3	Microtubule-associated protein 1A/1B-light chain 3
LC3II	LC3-phosphatidylethanolamine conjugate
LD	lipid droplet
LDL	Low-density lipoprotein
Mdivi-1	Mitochondrial division inhibitor
MDV	Mitochondria-derived vesicles
MFF	Mitochondrial fission factor
MSC	Mesenchymal stem cells
mtDNA	Mitochondrial DNA
mTORC1	Mammalian target of rapamycin complex 1
mtROS	Mitochondrial reactive oxygen species
NADPH	Nicotinamide adenine dinucleotide phosphate
NOX4	NADPH oxidase 4
Noxa	Phorbol-12-myristate-13-acetate-induced protein 1
NR4A1	Nuclear receptor subfamily 4 group A member 1
Nrf/ARE	Nuclear factor erythroid 2-related factor 2/antioxidant responsive element
P53	Tumor protein p53
PAD	Peripheral artery disease
PC12	Cell line derived from pheochromocytoma of the rat adrenal medulla
PGC1α	Peroxisome proliferator-activated receptor-γ coactivator 1-α
PIK3C3/Vps34	Phosphatidylinositol 3-kinase catalytic subunit type 3
PIK3R4/p150	Phosphoinositide 3-kinase regulatory subunit 4
PINK1	PTEN-induced kinase 1
PKCβ2	Protein Kinase C-β
PLIN2	Perilipin 2
PRAS40	Proline-rich Akt substrate of 40 kDa
PtdIns3K	Phosphatidylinositol 3-kinase complex
PtdIns3P	Phosphatidylinositol 3-phosphate
REDD1	Regulated in development and DNA damage responses 1
ROS	Reactive oxygen species
SH3GLB1	SH3 Domain Containing GRB2 Like, Endophilin B1
SNAP29	Synaptosome Associated Protein 29
SNARE	SNAP receptor
STX17	Syntaxin 17
T2D	Type-2-diabetes
TB1	Tat-Beclin1
TFAM	Mitochondrial transcription factor A
TFEB	Transcription Factor EB
TOMM20	Translocase Of Outer Mitochondrial Membrane 20
TSC1/2 complex	Tuberous sclerosis 1/2 complex
TUDCA	Tauroursodeoxycholic acid
TXNIP	Thioredoxin-interacting protein
ULK1	Unc-51 Like Autophagy Activating Kinase 1
USP30	Ubiquitin specific peptidase 30
UVRAG	UV radiation resistance-associated gene protein
VPS34	vacuolar sorting 34 protein
VAM7	Vacuolar morphogenesis protein 7
VAM9	Vacuolar morphogenesis protein 9
VAMP8	Vesicle-associated membrane protein 8
vWF	Von Willebrand factor
ZKSCAN3	Zinc Finger with KRAB And SCAN Domains 3
